# Remotely Assessing Fraction of Photosynthetically Active Radiation (*FPAR*) for Wheat Canopies Based on Hyperspectral Vegetation Indexes

**DOI:** 10.3389/fpls.2018.00776

**Published:** 2018-06-07

**Authors:** Changwei Tan, Dunliang Wang, Jian Zhou, Ying Du, Ming Luo, Yongjian Zhang, Wenshan Guo

**Affiliations:** Jiangsu Key Laboratory of Crop Genetics and Physiology, Jiangsu Co-Innovation Center for Modern Production Technology of Grain Crops, Joint International Research Laboratory of Agriculture and Agri-Product Safety of the Ministry of Education of China, Yangzhou University, Yangzhou, China

**Keywords:** hyperspectral vegetation index, wheat canopy, FPAR, assessment model, saturation

## Abstract

Fraction of photosynthetically active radiation (FPAR), as an important index for evaluating yields and biomass production, is key to providing the guidance for crop management. However, the shortage of good hyperspectral data can frequently result in the hindrance of accurate and reliable FPAR assessment, especially for wheat. In the present research, aiming at developing a strategy for accurate FPAR assessment, the relationships between wheat canopy FPAR and vegetation indexes derived from concurrent ground-measured hyperspectral data were explored. FPAR revealed the most strongly correlation with normalized difference index (NDI), and scaled difference index (N^*^). Both NDI and N^*^ revealed the increase as the increase of FPAR; however, NDI value presented the stagnation as FPAR value beyond 0.70. On the other hand, N^*^ showed a decreasing tendency when FPAR value was higher than 0.70. This special relationship between FPAR and vegetation index could be employed to establish a piecewise FPAR assessment model with NDI as a regression variable during FPAR value lower than 0.70, or N^*^ as the regression variable during FPAR value higher than 0.70. The model revealed higher assessment accuracy up to 16% when compared with FPAR assessment models based on a single vegetation index. In summary, it is feasible to apply NDI and N^*^ for accomplishing wheat canopy FPAR assessment, and establish an FPAR assessment model to overcome the limitations from vegetation index saturation under the condition with high FPAR value.

## Introduction

Fraction of photosynthetically active radiation (FPAR) absorbed by crops, as the fraction of incoming solar radiation in the spectral range of 400–700 nm absorbed by crop canopies (Moreau and Li, 1996; Ma et al., 2007), was critical to understanding and quantifying the exchange of mass, energy and momentum between atmosphere and land surface, which played an important role in most ecosystem productivity including crop biomass models (Muñoz et al., 2010). As the measurement parameter of the photosynthetic capacity of plant canopies linked to productivity, FPAR was beneficial to provide the guidance for crop cultivation activities (Vepsäläinen et al., 2004). However, conventional methods of FPAR assessment from field observation, with the involvement of site-specific complicated parameterizations and calculations, were difficult to apply in large agricultural areas. These shortcomings could be overcome through the complementary application of hyperspectral measurements with several advantages including non-destructive and uniform operation, and rapid accomplishment without complicated parameterizations for crops.

FPAR assessment from vegetation indexes (VIs) obtained from hyperspectral data, especially remote sensing data, had been reported by several previous studies (Fensholt et al., 2004; Fensholt, 2006; Olofsson et al., 2007; Zhao et al., 2009; Chiesi et al., 2011). Through comparing FPAR assessment of legume crops, among nine kinds of VIs with the close relationship with FPAR, modified soil-adjusted vegetation index (MSAVI) had the best VI performance (Ridao et al., 1998). On the other hand, ground cover could result in the significant reduction of background impact so that FPAR could be better assessed using normalized difference vegetation index (NDVI). Re-normalized difference vegetation index (RDVI) had demonstrated an approximate linear correlation with FPAR regardless of ground cover. Hyperspectral remote sensing was an important technique to fulfill real-time monitoring for growth status of crops based on its superior performance in acquiring vegetation canopy information rapidly and non-destructively. However, it still had the statistical uncertainty using regression analysis based on only five points. Other studies using radiation transfer models showed a linear relationship and conclude that NDVI had the better performance for FPAR assessment (Goward and Huemmrich, 1992; Hu et al., 2007; Yang et al., 2014).

Recently, modeled FPAR products based on MODIS had been reported in several studies. The increasing availability of time series of FPAR derived from MODIS had been investigated to confirm the significant change of three dynamic habitat index components in their magnitude, which was due to the larger MODIS FPAR than Medium Resolution Imaging Spectrometer (MERIS) FPAR (Coops et al., 2010). Previous reports had also conducted the comparison between MODIS FPAR and on-site measurements in USA to reveal the overestimation of ground-measured FPAR. The FPAR from MODIS with in-situ measurements in a tropical rainforest in Brazil had been compared to obtain a conclusion that MODIS FPAR was reliable for FPAR assessment (Turner et al., 2005). However, the evaluation of VI performance in different vegetation ecosystems was highly necessary (Olofsson et al., 2007).

Models based on linear FPAR-NDVI relationships suffered from a major flaw with the saturation of NDVI at the higher leaf area index (~3.5) (Samanta et al., 2012), thereby resulting in the lower sensitivity to FPAR change using a linear model in such case (Myneni and Williams, 1994; Zhang et al., 2009). Another issue was the limited data for boreal ecosystems. Meanwhile, the empirical evidence with a relationship between FPAR and hyperspectral VIs based on the major focus on forests, grasses (prairies), and some types of crops such as rice, wheat and cotton had also been confirmed by above mentioned studies (Yang et al., 2014). In contrast, there were few reports on quantitatively assessing FPAR for wheat canopies using VI from remote sensing data (Wang et al., 2016). Furthermore, existing remote sensing-based FPAR products lacked adequate ground validation critical for confirming the uncertainty and accuracy, therefore, these products could not be used for guiding crop production practice (Tan et al., 2013). According to above results, narrow-band hyperspectral data was massive, and could be obtained rapidly and non-destructively, and had a positive relationship with crop FPAR, so it was feasible to assess the FPAR with narrow-band hyperspectral data.

In the present research, in order to develop a practical methodology for assessing FPAR of wheat canopies, exhaustive statistical analysis of FPAR-VI relationships for wheat canopies was conducted by using ground-measured hyperspectral data collected from a series of field experiments.

## Materials and methods

### Experimental design

Four wheat variants including Yangmai 13, Yangmai 15, Yangmai 16, and Ningmai 9 were used during March to May of 2015–2017 in the experimental field of Yangzhou University, China (119°18′E, 32°26′N). Prior to experiments, the layer of 0–30 cm in yellow brown soil (Alfisolsin U.S. taxonomy) with previous plantation of rice contained 121.4 mg·kg^−1^ nitrogen, 25.9 mg·kg^−1^ phosphorus, 83.7 mg·kg^−1^ potassium and 2.19% organic matter. Canopy hyperspectral measurements coupled with quasi-simultaneous measurements of photosynthetically active radiation (PAR) during the growth of wheat canopies were conducted. In order to highlight the variations during wheat growth due to biochemical composition changes, three different levels of nitrogen fertilization (urea) including non-nitrogen fertilization, adequate nitrogen fertilization (450 kg.ha^−1^) and heavy nitrogen fertilization (900 kg·ha^−1^) were implemented. The experiments were conducted in triplicate for each nitrogen level. The dimension of the plot was 20 × 20 m. Local standards for wheat cropping management practices to control water, pest, disease and weed should be abided. Training data consisted of 95 samples and 87 samples from 2015 and 2016, and test data comprised 50 samples from 2017.

### Canopy hyperspectral reflectance measurement

In 2015, six hyperspectral measurements were carried out at the wheat turning green stage (March 7), jointing stage (March 20), booting stage (April 9), blooming stage (April 25), 15 days after blooming stage (May 9), and milking stage (May 18), respectively. All spectrometric determinations of the canopy were conducted from a vertical height to wheat canopies of 1.6 m, under cloudless or nearly cloudless condition between 11:00 and 14:00, employing an ASD FieldspecPro spectrometer (Analytical Spectral Devices, Boulder, CO, USA) equipped with 25° field of view fiber optics through operating in 350–2,500 nm hyperspectral region with a sampling interval of 1.4 nm between 350 and 1,050 nm, and 2 nm between l,050 and 2,500 nm, and with a spectral resolution of 3 nm at 700 nm, 10 nm at 1,400 nm, selecting a representative, uniform growth, pest-free plants, and the sensor probe during the measurement. A 40 × 40 cm BaSO_4_ calibration panel was employed for the calculation of hyperspectral reflectance. Crop and panel luminance measurements were conducted with mean scanning of 20 times at optimal integration time, with a dark current correction at each spectrometric determination.

In 2016, four hyperspectral measurements of 87 samples were performed at wheat turning green stage (March 9), jointing stage (March 22), blooming stage (April 23), and milking stage (May 20), respectively. The others were same as that in 2015.

In 2017, three hyperspectral measurements of 50 samples were performed at wheat booting stage (April 11), blooming stage (April 22), and 15 days after blooming stage (May 12), respectively. The others were same as that in 2015.

### Hyperspectral smoothing

In order to eliminate high frequency noise and the random errors from hyperspectral measurement instruments, a hyperspectral smoothing process was conducted to improve signal-noise ratio. A five-point weighted smoothing method was used to process the raw hyperspectral data (Smith et al., 2004). Five-point weighted smoothing method was calculated based on Equation (1):

$$\begin{array}{r} n{=}^{\left(\frac{m_{-2}}{4}+\frac{m_{-1}}{2}+\frac{m}{1}+\frac{m_{1}}{2}+\frac{m_{2}}{4}\right)}{/}_{25} \end{array}$$

Here, *n* represented the weighted average of the intermediate data points in the filter window, namely the smoothed hyperspectral value, and *m* represented the value of unsmoothed data points, namely the raw hyperspectral value.

### FPAR measurement

All PAR measurements were conducted to synchronize with canopy spectrometric determinations, with the same target as spectroscopic measurement, using LI-191SA line quantum sensor produced by American LI-COR Company. The instrument's light quantum sensing area was 1 m × 12.7 mm, the sensing wavelength was 400–700 nm, the measured result was the average PAR within the scope of sensing area, and the output unit was μmol.m^−2^·s^−1^. The measured target included four fractions of PAR: PAR canopy incident (PARci), PAR canopy reflection (PARcr), PAR ground incident (PARgi), and PAR ground reflection (PARgr). Top-of-canopy measurements were conducted by placing the linear quantum sensor above the canopy of 0.5 m. Under-canopy measurements were conducted above the ground of 0.15 m with the aim of both ends of the probe sensing part at middle position between rows and the probe midpoint at the top of plant row, thus enabling the horizontal ball to stay on the midpoint of spirit level, and the linear quantum sensor at the horizontal level.

The (canopy-) absorbed PAR (APAR) could be estimated by subtracting PAR reflected to atmosphere and PAR absorbed by soil from total incident PAR. Therefore, FPAR was calculated using Equation (2) (Ridao et al., 1998):

$$\begin{array}{r} FPAR{=}^{\left[PAR_{ci}-PAR_{cr}-\left(PAR_{gi}-PAR_{gr}\right)\right]}{/}_{PAR_{ci}} \end{array}$$

Here, PARci was photosynthetic active radiation canopy incident, PARcr was photosynthetic active radiation canopy reflection, PARgi was photosynthetic active radiation ground incident, and PARgr was photosynthetic active radiation ground reflection.

### Hyperspectral vegetation indexes and analysis method

According to previous studies (Tan et al., 2013) and hyperspectral characteristics of wheat combined with a physical significance of hyperspectral indexes, many VIs can be used to assess crop FPAR. however, there is not uniform VI to assess crop FPAR. Here, a total of 54 VIs (Table 1) related to FPAR, leaf area index and chlorophyll (known as an important impact on PAR absorbed by green vegetation) as the independent variables for establishing remote sensing assessment models of wheat canopy FPAR were considered. Data derived from the experimental field in 2015 (95 samples) and 2016 (87 samples) were employed to establish the regression models, and data derived from the experimental field in 2017 (50 samples) were employed to validate the models.

**Table 1:** Hyperspectral vegetation indexes (VIs) used in the research.

**Vegetation index**	**Abbreviation**	**Algorithm**	**Source**
Simple ratio 1	SR[787, 765]	R_787_/R_765_	Stenberg et al., 2004
Simple ratio 2	SR[415, 710]	R_415_/R_710_	Stenberg et al., 2004
Simple ratio 3	SR[415, 695]	R_415_/R_695_	Stenberg et al., 2004
Simple ratio 4	SR[750, 705]	R_750_/R_705_	Stenberg et al., 2004
Simple ratio 5	SR[900, 680]	R_900_/R_680_	Stenberg et al., 2004
Simple ratio 6	SR[801, 670]	R_801_/R_670_	Stenberg et al., 2004
Simple ratio 7	SR[672, 550, 708]	R_672_/(R_550_^*^ R_708_)	Stenberg et al., 2004
Optimized vegetation index 1	VIopt1	R_760_/R_730_	Stenberg et al., 2004
Optimized vegetation index 2	VIopt2	100 ^*^ (lnR_760_- lnR_730_)	Stenberg et al., 2004
Pigment specific simple ratio 1	PSSR[800, 680]	R_800_/R_680_	Stenberg et al., 2004
Pigment specific simple ratio 2	PSSR[800, 635]	R_800_/R_635_	Stenberg et al., 2004
Pigment specific simple ratio 3	PSSR[800, 470]	R_800_/R_470_	Stenberg et al., 2004
Zarco-Tejada and Miller	ZTM	R_750_/R_710_	Stenberg et al., 2004
Red-edge model index	R-M	(R_750_/R_720_)−1	Gobron et al., 2000
Difference index	DI	R_800_-R_550_	Gobron et al., 2000
Difference vegetation index	DVI	R_800_-R_680_	Gobron et al., 2000
Pigment specific normalized difference 1	PSND[800, 635]	(R_800_-R_635_)/(R_800_ + R_635_)	Bargain et al., 2013
Pigment specific normalized difference 2	PSND[800, 470]	(R_800_-R_470_)/(R_800_ + R_470_)	Bargain et al., 2013
Modified simple ratio index 1	mSRI1	(R_750_-R_445_)/(R_705_+ R_445_)	Bargain et al., 2013
Modified simple ratio 2	mSRI2	(R_800_/R_670_-1)/SQRT(R_800_/R_670_ + 1)	Gonzalezdugo et al., 2015
Normalized difference index	NDI	(R_800_-R_680_)/(R_800_+R_680_)	Gonzalezdugo et al., 2015
Modified normalized difference index	mNDI	(R_750_-R_705_)/(R_750_+R_705_-2^*^R_445_)	Fassnacht et al., 2015
Plant senescence reflectance index	PSRI	(R_680_-R_500_)/R_750_	Fassnacht et al., 2015
Re-normalized difference vegetation index	RDVI	(R_800_-R_670_)/SQRT(R_800_ + R_670_)	Skakun et al., 2003
Simple ratio pigment index	SRPI	R_430_/R_680_	Griend and Owe, 1993
Ratio vegetation index	RVI	(R_790_:R_810_)/(R_640_:R_660_)	Ridao et al., 1998
Normalized pigments chlorophyll ratio index	NPCI	(R_680_-R_430_)/(R_680_ + R_430_)	Ridao et al., 1998
Normalized phaeophytinization index	NPQI	(R_415_-R_435_)/(R_415_ + R_435_)	Ridao et al., 1998
Structure intensive pigment index	SIPI	(R_800_-R_445_)/(R_800_- R_680_)	Chen, 2014
MERIS terrestrial chlorophyll index	MTCI	(R_750_-R_710_)/(R_710_ - R_680_)	Chen, 2014
Modified chlorophyll absorption in reflectance index	MCARI	[(R_700_-R_670_)−0.2 ^*^ (R_700_-R_550_)] ^*^ (R_700_/R_670_)	Major et al., 1990
Green normalized difference vegetation index	GNDVI	(R_800_-R_550_)/(R_800_ + R_550_)	Wang et al., 2009
Modified transformed vegetation index	MTVI	1.2 ^*^ [1.2 ^*^ (R_800_- R_550_)−2.5 ^*^ (R_670_-R_550_)]	Huggins et al., 2017
Photochemical reflectance index	PRI	(R_531_-R_570_)/(R_530_ + R_570_)	Dash and Curran, 2004
Transformed vegetation index	TVI	0.5 ^*^ [120 ^*^ (R_750_- R_550_)−200 ^*^ (R_670_-R_550_)]	Kimura et al., 2004
Temperature condition index	TCI	1.2 ^*^ (R_700_-R_550_)−1.5 ^*^ (R_670_-R_550_) ^*^ SQRT(R_700_/R_670_)	Fu et al., 2015
Double difference index	DDI	(R_750_-R_720_)–(R_700_-R_670_)	Rondeaux et al., 1996
Scaled difference index	N*	(NDVI–NDVI_0_)/(NDVI_S_-NDVI_0_)	Barton and North, 2001
Modified soil adjusted vegetation index	MSAVI	0.5 ^*^ [2 ^*^ R_800_ + 1–SQRT((2 ^*^ R_800_ + 1)∧2–8 ^*^ (R_800_-R_670_))]	Skianis et al., 2007
Optimal soil adjusted vegetation index	OSAVI	(1 + 0.16)^*^(R_800_-R_670_)/(R_800_ + R_670_ + 0.16)	Skianis et al., 2007
Transformed chlorophyll absorption in reflectance index	TCARI	3 ^*^ [(R_700_- R_670_)−0.2 ^*^ (R_700_-R_550_) ^*^ (R_700_/R_670_)]	Guo et al., 2015
Visible atmospherically resistant index	VARI	(R_555_-R_680_)/(R_555_ + R_680_-R_480_)	Qi et al., 1994
Wide dynamic range vegetation index	WDRVI	(α^*^R_nir_-R_red_)/(α^*^R_nir_ + R_red_), a = 0.05, 0.1, 0.2	Steven, 1998
Red green ratio	RGR	(R_612_ + R_660_)/(R_510_ + R_560_)	Steven, 1998
Normalized difference vegetation index 1	NDVI[760, 708]	(R_760_-R_708_)/(R_760_ + R_708_)	Xiao et al., 2014
Normalized difference vegetation index 2	NDVI[800, 600]	(R_800_- R_600_) / (R_800_ + R_600_)	Xiao et al., 2014
Normalized difference vegetation index 3	NDVI[780, 550]	(R_780_-R_550_)/(R_780_ + R_550_)	Xiao et al., 2014
Normalized difference vegetation index 4	NDVI[800, 700]	(R_800_-R_700_)/(R_800_ + R_700_)	Xiao et al., 2014
Normalized difference vegetation index5	NDIV[900, 680]	(R_900_-R_680_)/(R_900_ + R_680_)	Xiao et al., 2014
Ratio between TCI and OSAVI	TCI/OSAVI	TCI/OSAVI	Kaiser, 2005
Ratio between MTVI and MSAVI	MTVI/MSAVI	MTVI/MSAVI	Kaiser, 2005
Ratio between DDI and MSAVI	DDI/MSAVI	DDI/MSAVI	Kaiser, 2005
Ratio between MCARI and OSAVI	MCARI/OSAVI	MCARI/OSAVI	Kaiser, 2005
Ratio between TCARI and OSAVI	TCARI/OSAVI	TCARI/OSAVI	Kaiser, 2005

*R indicated hyperspectral reflectance of crops*.

VI-FPAR relationships were analyzed using a variety of regression models such as linear, exponential, logarithmic, and quadratic models. Models were ranked based on statistically significant (*p* < 0.05 or 0.001) correlation coefficients (*r* in case of linear models) and coefficients of determination (*R*^2^ in case of non-linear models). Finally, after making a schematic plot to describe the relationship between estimated and measured FPAR values under the scale of 1:1, the model performance was validated by using determination coefficients (*R*^2^) and relative root mean squared error (RRMSE) for the assessment of ground-measured FPAR. The greater *R*^2^ and the less RRMSE could result in the higher precision of the model to assess FPAR of wheat canopies. According to Equations (3, 4), respectively, the RRMSE and assessment accuracy were carried out as follows:

$$\begin{array}{r} RRMSE{=}^{\sqrt{\frac{1}{n}\overset{n}{\underset{i=1}{\sum }}{\left(y_{i}-{ŷ}_{i}\right)}^{2}}}{/}_{\frac{1}{n}\overset{n}{\underset{i=1}{\sum }}y_{i}} \end{array}$$

$$\begin{array}{r} \text{Assessmentaccuracy}=1-\text{RRMSE} \end{array}$$

Here, *y*_*i*_ and ŷ_*i*_ represented the measured values and predicted values of wheat canopy FPAR, respectively. *n* was the number of samples.

## Results

### Changes in wheat canopy FPAR with growth stage

**Figure 1:**
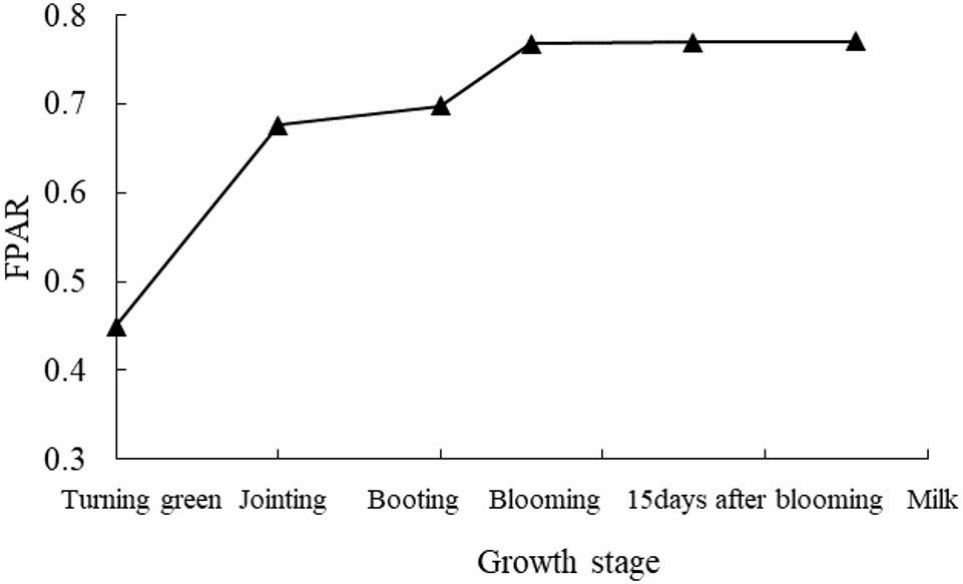
Changes of wheat canopy FPAR at different growth stages.

FPAR revealed the progressive increase as the growth of wheat crops at different growth stages (Figure 1). An initial large increase in FPAR, by approximately 50.6%, was observed corresponding to crop development from turning stage to jointing stage. Further increase in FPAR, from booting stage to milk stage, was observed at lower rates of 3.1, 10.1, 1.17, and 1.30%, respectively. Once at blooming stage, the increase of FPAR was initiated until FPAR reached up to the maximum value of 0.76. From blooming stage to milk stage, FPAR revealed a tendency of slow increase or reached the saturation status.

### VI-FPAR relationships

Statistically significant correlations between FPAR and VIs with both positive and negative correlations were observed in 51 cases out of 54 VIs considered (Table 2). Positive correlations between VIs and FPAR were generally stronger than negative ones. FPAR revealed the strongest correlation with NDI, N^*^, and NDVI [760, 708] with *r* of 0.902, 0.884, and 0.853, respectively. Therefore, NDI, N^*^ and NDVI [760, 708] could be confirmed as common VIs relatively well correlated to wheat canopy FPAR, which were the probable VI choices for assessing wheat canopy FPAR.

**Table 2:** Linear relationships between wheat canopy FPAR and VIs.

**Vegetation index**	***R***	**Vegetation index**	***R***	**Vegetation index**	***r***
SR[787, 765]	0.814^++^	mSRI2	0.783^++^	MSAVI	0.841^++^
SR[415, 710]	0.437^+^	NDI	0.902^++^	OSAVI	0.739^++^
SR[415, 695]	−0.514^++^	Mndi	0.637^++^	VARI	0.531^++^
SR[750, 705]	0.631^++^	PSRI	−0.786^++^	TCARI	−0.103
SR[900, 680]	0.617^++^	RDVI	0.782^++^	WDRVI(a = 0.05)	0.761^++^
SR[801, 670]	0.734^++^	SRPI	0.348^+^	WDRVI(a = 0.1)	0.784^++^
SR[672,550, 708]	0.684^++^	RVI	0.703^++^	WDRVI(a = 0.2)	0.735^++^
VIopt1	0.784^++^	NPCI	−0.386^+^	RGR	−0.577^++^
VIopt2	0.806^++^	NPQI	−0.583^++^	NDVI[760, 708]	0.853^++^
PSSR[800, 680]	0.657^++^	SIPI	−0.781^++^	NDVI[800, 600]	0.836^++^
PSSR[800, 635]	0.781^++^	MTCI	0.573^++^	NDVI[780, 550]	0.846^++^
PSSR[800, 470]	0.764^++^	MCARI	0.347^+^	NDVI[800, 700]	0.832^++^
ZTM	0.571^++^	GNDVI	0.832^++^	NDIV[900, 680]	0.841^++^
R-M	0.471^++^	MTVI	0.682^++^	TCI/OSAVI	−0.097
DI	0.731^++^	PRI	0.519^++^	MTVI/MSAVI	0.633^++^
DVI	0.697^++^	TVI	0.468^++^	DDI/MSAVI	0.302^+^
PSND[800, 635]	0.831^++^	TCI	−0.197	MCARI/OSAVI	0.104
PSND[800, 470]	0.847^++^	DDI	0.418^++^	TCARI/OSAVI	0.0103
mSRI1	0.771^++^	N^*^	0.884^++^	−	−

^+^ *and ^++^represented significant difference at probability levels of 0.05 and 0.01, respectively*.

### Establishment of FPAR assessment model based on VI

**Figure 2:**
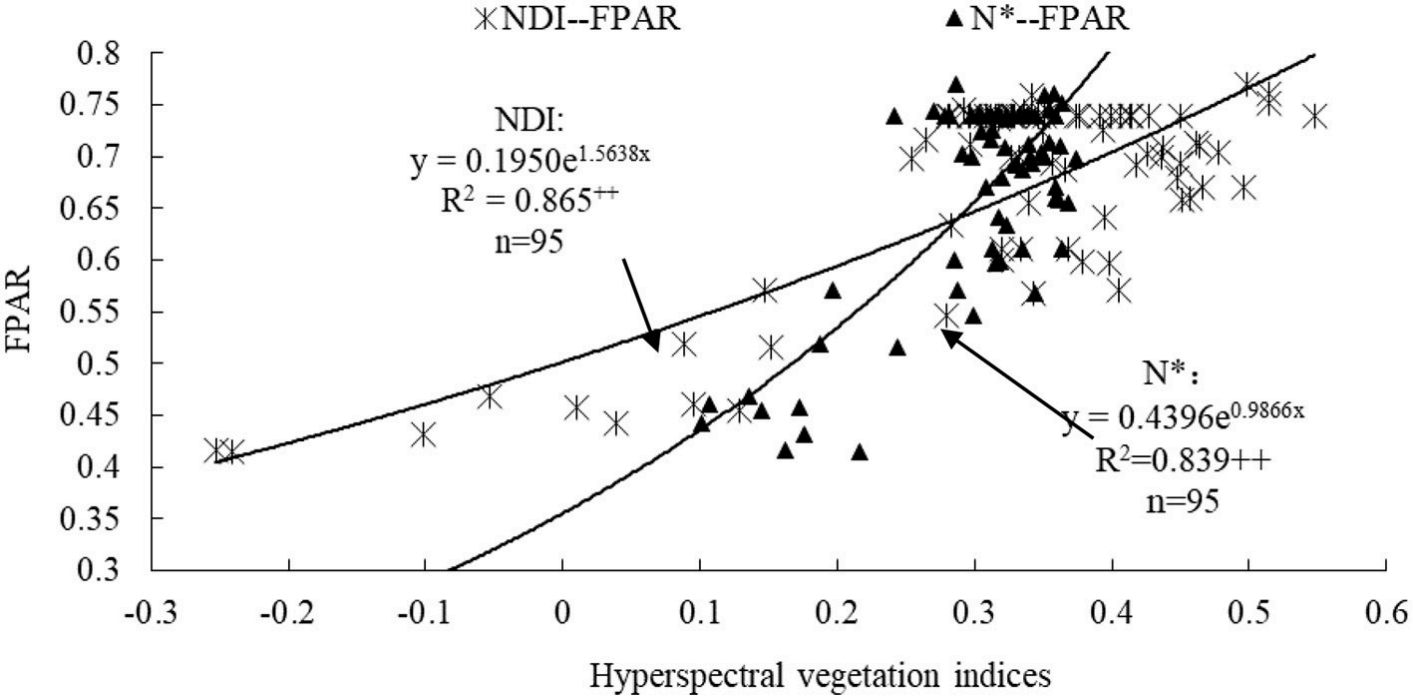
NDI-FPAR and N^*^-FPAR relationships for wheat canopies. ++ represented significant difference at the probability level of 0.01.

A total of 12 VIs were considered for modeling FPAR based on a threshold on VI-FPAR correlation (*r* > 0.81 in Table 1). These non-linear FPAR assessment models were best represented as exponential functions, and evaluated using their predictive (*R*^2^) and error statistics (RRMSE) (Table 3). Among them, FPAR revealed the strongest exponential relationship with NDI, and a stronger exponential relationship with N^*^ and NDVI [760, 708]. In addition, the models using NDI, N^*^ and NDVI [760, 708] as the variables had good performance to accomplish FPAR assessment with *R*^2^ of 0.937, 0.891, and 0.886, with RRMSE of 0.114, 0.143, and 0.152, and with assessment accuracy of 88.6, 85.7, and 84.8%, respectively. Furthermore, according to comparisons among *R*^2^, RRMSE and assessment accuracy, it was more suitable to assess wheat canopy FPAR using NDI and N^*^ (Figure 2) than using NDVI [760, 708].

**Table 3:** Quantitative relationships between wheat canopy FPAR (y) and VI (x).

**Hyperspectral vegetation index**	**Model**	***R^2^***	**RRMSE**
SR[787, 765]	*y* = 0.0175e^2.4096x^	0.764^++^	0.163
PSND[800, 635]	*y* = 0.1797e^1.8565x^	0.757^++^	0.167
PSND[800, 470]	*y* = 0.0470e^2.6013x^	0.764^++^	0.159
NDI	*y* = 0.1950e^1.5638x^	0.865^++^	0.114
GNDVI	*y* = 0.1756e^1.4955x^	0.772^++^	0.161
N^*^	*y* = 0.4396e^0.9866x^	0.839^++^	0.143
MSAVI	*y* = 0.0247e^2.5335x^	0.762^++^	0.177
NDVI[760, 708]	*y* = 0.2746e^1.0757x^	0.822^++^	0.176
NDVI[800, 600]	*y* = 0.1637e^1.7319x^	0.779^++^	0.161
NDVI[780, 550]	*y* = 0.1396e^1.7401x^	0.735^++^	0.152
NDVI[800, 700]	*y* = 0.2003e^0.9769x^	0.751^++^	0.159
NDIV[900, 680]	*y* = 0.1946e^0.8476x^	0.736^++^	0.172

++ *represented significant difference at 0.01 level (P < 0.01)*.

### Saturation analysis of vis

**Figure 3:**
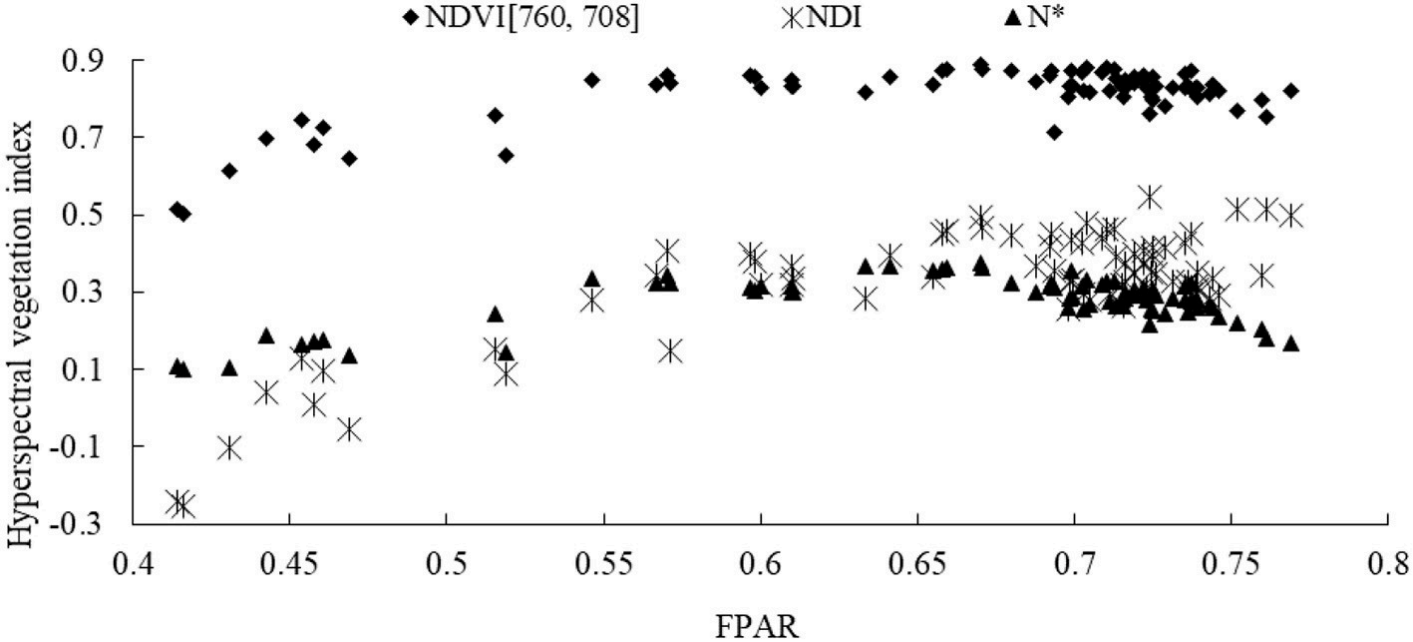
Changes of NDI, N^*^ and NDVI[760, 708] with wheat canopy FPAR (*n* = 87).

Three VIs including NDI, N^*^ and NDVI [760, 708] shown in Figure 3 revealed the strongest relationship with FPAR, and progressive increase as the increase of FPAR up to 0.70. The continuous increase of NDI and NDVI [760, 708] was terminated at FPAR values of 0.68 and 0.70, suggesting the saturation status. On the other hand, N^*^ displayed a different trend evidenced the continuous decrease in spite of FPAR value of 0.70, indicating that a reliable FPAR model could be established using NDI as the predictor when the saturation point was reached at FPAR lower than 0.70, and using N^*^ as the predictor when the saturation point was reached at FPAR higher than 0.70, which will effectively address the issue of VI saturation.

**Figure 4:**
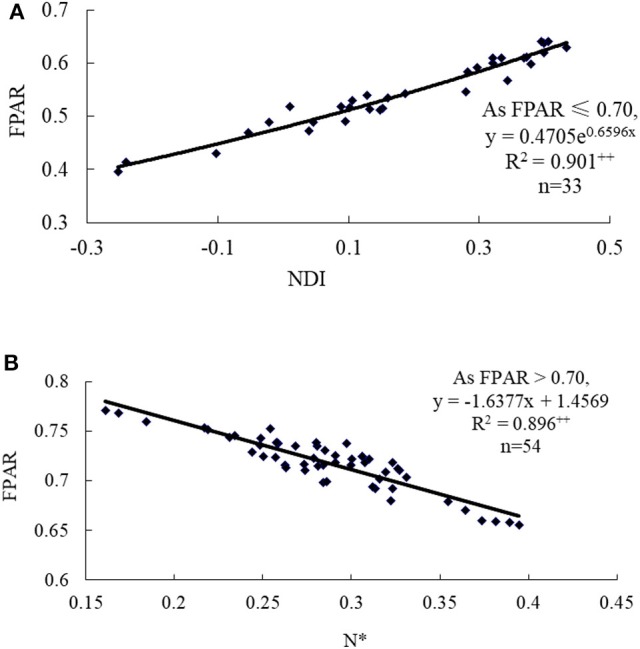
Hyperspectral VI-based assessment models such as **(A)** NDI-FPAR model (FPAR ≤ 0.70) and **(B)** N^*^-FPAR model (FPAR > 0.70) for wheat canopy FPAR. ++ represented significant difference at the probability level of 0.01.

According to the range of FPAR values from the aforementioned results, the segmented hyperspectral FPAR assessment model was also constructed, as shown in Figure 4. Namely, if FPAR was < 0.70, NDI should be used to assess FPAR, and the monitoring model was y = 0.4705e^6596x^, *R*^2^ = 0.901 (*p* < 0.01); if FPAR was higher than 0.70, N^*^ should be used to assess FPAR, and the assessment model was y = −1.6377x +1.4569, *R*^2^ = 0.796 (*p* < 0.01).

### Evaluation of VI-based FPAR model

**Figure 5:**
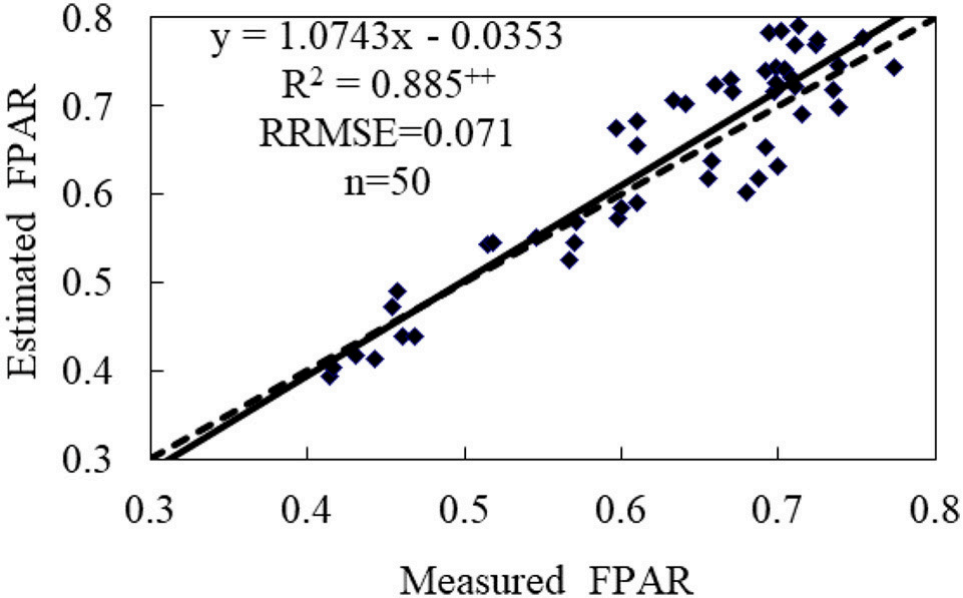
Evaluating the assessment capability of the piecewise model for wheat canopy FPAR. ++ represented significant difference at 0.01 level. The solid and dashed lines were the actual and 1:1 relationship between estimated and measured FPAR values, respectively.

Totally 50 samples collected from the experiment in 2017 were employed to validate the hyperspectral VI-based FPAR assessment model. The predicted and measured FPAR cross-resistance was nearly coincided with 1:1 relationship line, as shown in Figure 5. Under the condition with low FPAR values, the estimated value may be underestimated. As the increase of FPAR, the predicted values will be closer to the measured values with *R*^2^, RRMSE and assessment accuracy of 0.865, 0.071, and 92.9% for the piecewise FPAR model, respectively. Compared with the assessment models based on NDI, N^*^ and NDVI [760, 708] alone in Table 3, the assessment accuracy of the piecewise FPAR model in different FPAR ranges revealed the increase by 11.3, 13.9, and 16.4%, respectively. In summary, the piecewise model based on NDI and N^*^ for assessing FPAR can not only enhance the assessment accuracy, but also solve saturation problems of NDI and NDVI [760, 708].

## Discussion

FPAR is mainly controlled by ground cover and leaf area. Before jointing stage, FPAR presents a significant increase (Figure 1), which is characterized by strong absorption of incoming PAR as the vigorous growth of wheat crops with improved leaf area driven by nitrogen fertilization. Sequentially, a lower rate of crop growth or leaf area expansion is followed due to the lower increasing rate of FPAR. According to agronomic principle of wheat, although FPAR data is limited after milk stage, a conclusion that wheat leaves start to change yellow and gradually litter as the extension of growth process, and FPAR exhibits the decline with the accompany of wheat's photosynthetic physiological characteristics still could be drawn. Until fully ripen stage, FPAR is close to 0, because wheat leaves take off green and become withered and even death so that they are unable to absorb light energy.

Currently, many efforts are focusing on the application of VIs, especially NDVI, for assessing crop canopy FPAR. Furthermore, many studies have documented that VIs are better correlated with FPAR than the reflectance in a single waveband (Musick and Pelletier, 1988; Read et al., 2002), which could be, plausibly, explained by the fact that VIs can reduce the impact from atmospheric scattering and soil background to the minimal level with the obviously enhanced wavelength sensitivity (Huete et al., 2002). Similarly, in our study, FPAR is highly correlated with the majority of VIs (49 out of 54), with the best performance from NDI, N^*^ and NDVI [760, 708], which is helpful to provide an important technique for the establishment of perfect wheat photosynthetic groups, the improvement of sunlight energy efficiency, and the implementation of cultivation control.

Hyperspectral technology is widely used in precision agriculture because it can rapidly, non-destructively and accurately monitor crop growth information. It is of great significance for improving the accuracy of FPAR estimation with hyperspectral parameters. As compared with previous studies using NDVI, NDI and N^*^ for assessing FPAR, our study has the lower RRMSE and higher assessment accuracy, and the utilization model is a function of FPAR and VI. This provides a theoretical basis for the development of ecological process models in crop. It is considered as a health indicator for crop growth, and help to work out some feasible plans for crop planting. Therefore, hyperspectral monitoring of FPAR plays an important role in crop production management and precision agriculture. Future study should aim at assessing the performance of the proposed model during the growth of wheat under various conditions, even different wheat variants and other types of crops. Meanwhile, this study will also be helpful for refining the model as a useful tool for informing crop management practices. More efforts should also be made to test this model with data from different sources including field-based hyperspectral measurements and current and future satellite data.

VIs including NDVI are often plagued with saturation in high biomass areas as a major disadvantage for VI-FPAR models. We have addressed this issue by employing the differences in the sensitivity of different VIs to FPAR. When FPAR is higher than 0.70, NDI and NDVI [760, 708] tend to termination, but N^*^ shows a declining trend. Accordingly, our proposed piecewise FPAR model uses NDI (FPAR ≤ 0.70), or N^*^ (FPAR > 0.70) as the indicator. Given that VIs can be assessed consistently from both field-based hyperspectral data and satellite data, this model can also be used for either data source, which will provide a useful resource for our model using not only the current satellite data, but also the data from future satellite sensors (such as Hys PIRI).

## Author contributions

CT and WG conceived the research. CT, DW, and JZ designed and performed the experiments. CT, DW, and YD prepared and revised the manuscript. CT, ML, and DW analyzed the data. DW, YD, and YZ provided technical.

### Conflict of interest statement

The authors declare that the research was conducted in the absence of any commercial or financial relationships that could be construed as a potential conflict of interest.

## References

[B1] Bargain A. Robin M. Méléder V. Rosa P. Menn E. L. Harin N. (2013). Seasonal spectral variation of Zostera noltii and its influence on pigment-based vegetation indices. J. Exp. Mar. Biol. Ecol. 446, 86–94. 10.1016/j.jembe.2013.04.012

[B2] Barton C. V. M. North P. R. J. (2001). Remote sensing of canopy light use efficiency using the photochemical reflectance index: model and sensitivity analysis. Remote Sens. Environ. 78, 264–273. 10.1016/S0034-4257(01)00224-3

[B3] Chen J. M. (2014). Evaluation of vegetation indices and a modified simple ratio for boreal applications. Can. J. Remote Sens. 22, 229–242. 10.1080/07038992.1996.10855178

[B4] Chiesi M. Fibbi L. Genesio L. Gioli B. Magno R. Maselli F. (2011). Integration of ground and satellite data to model mediterranean forest processes. Int. J. Appl. Earth Obs. 13, 504–515. 10.1016/j.jag.2010.10.006

[B5] Coops N. C. Michaud J. Andrew M. E. Wulder M. A. (2010). Comparison of a regional-level habitat index derived from MERIS and MODIS estimates of canopy-absorbed photosynthetically active radiation. Remote Sens. Lett. 2, 327–336. 10.1080/01431161.2010.516281

[B6] Dash J. Curran P. (2004). The MERIS terrestrial chlorophyll index. Int. J. Remote Sens. 25, 5403–5413. 10.1080/0143116042000274015

[B7] Fassnacht F. E. Stenzel S. Gitelson A. A. (2015). Non-destructive estimation of foliar carotenoid content of tree species using merged vegetation indices. J. Plant Physiol. 176, 210–217. 10.1016/j.jplph.2014.11.003 25512167

[B8] Fensholt R. (2006). Evaluation of fraction of absorbed photosynthetically active radiation products for different canopy radiation transfer regimes - methodology and results using joint research center products derived from SeaWIFS against ground-based estimations. J. Geophys. Res. 111, 2943–2979. 10.1029/2005JD006511

[B9] Fensholt R. Sandholt I. Rasmussen M. S. (2004). Evaluation of MODIS LAI, FAPAR and the relation between FAPAR and NDVI in a semi-arid environment using in-situ measurements. Remote Sens. Environ. 91, 490–507. 10.1016/j.rse.2004.04.009

[B10] Fu G. Shen Z. X. Zhong Z. M. (2015). Initial response of normalized difference vegetation index, green normalized difference vegetation index and soil adjusted vegetation index to infrared warming in highland barley of the tibet. Ecol. Environ. Sci. 24, 365–371.

[B11] Gobron N. Pinty B. Verstraete M. M. Widlowski J. L. (2000). Advanced vegetation indices optimized for up-coming sensors: design, performance, and applications. IEEE T. Geosci. Remote Sens. 38, 2489–2505. 10.1109/36.885197

[B12] Gonzalezdugo V. Hernandez P. Solis I. Zarcotejada P. (2015). Using high-resolution hyperspectral and thermal airborne imagery to assess physiological condition in the context of wheat phenotyping. Remote Sens. Basel. 7, 13586–13605. 10.3390/rs71013586

[B13] Goward S. N. Huemmrich K. F. (1992). Vegetation canopy PAR absorptance and the normalized difference vegetation index: an assessment using the sail model. Remote Sens. Environ. 39, 119–140. 10.1016/0034-4257(92)90131-3

[B14] Griend A. A. V. D. Owe M. (1993). On the relationship between thermal emissivity and the normalized difference vegetation index for natural surfaces. Int. J. Remote Sens. 14, 1119–1131. 10.1080/01431169308904400

[B15] Guo X. Zhang H. Yuan T. Zhao J. Xue Z. (2015). Detecting the temporal scaling behavior of the normalized difference vegetation index time series in china using a detrended fluctuation analysis. Remote Sens. Basel. 7, 12942–12960. 10.3390/rs71012942

[B16] Hu J. Su Y. Tan B. Huang D. Yang W. Schull M. (2007). Analysis of the MISR LAI/FPAR product for spatial and temporal coverage, accuracy and consistency. Remote Sens. Environ. 107, 334–347. 10.1016/j.rse.2006.06.020

[B17] Huete A. Didan K. Miura T. Rodriguez E. P. Gao X. Ferreira L. G. (2002). Overview of the radiometric and biophysical performance of the MODIS vegetation indices. Remote Sens. Environ. 83, 195–213. 10.1016/S0034-4257(02)00096-2

[B18] Huggins T. D. Mohammed S. Sengodon P. Ibrahim A. M. H. Tilley M. Hays D. B. (2017). Changes in leaf epicuticular wax load and its effect on leaf temperature and physiological traits in wheat cultivars (*Triticum aestivum L*.) Exposed to high temperatures during anthesis. J. Agron. Crop Sci. 71, 217–223. 10.1111/jac.12227

[B19] Kaiser J. (2005). Modis-derived visible atmospherically resistant index for monitoring chaparral moisture content. Int. J. Remote Sens. 26, 3867–3873. 10.1080/01431160500185342

[B20] Kimura R. Okada S. Miura H. Kamichika M. (2004). Relationships among the leaf area index, moisture availability, and spectral reflectance in an upland rice field. Agr. Water Manage. 69, 83–100. 10.1016/j.agwat.2004.04.009

[B21] Ma J. Y. Liu J. M. Li S. K. Liang H. Jiang C. Y. Wang B. Z. (2007). Study on the features of the photosynthetic active radiation (PAR) with experimentations and measurements. J. Nat. Resour. 22, 673–682.

[B22] Major D. J. Baret F. Guyot G. (1990). A ratio vegetation index adjusted for soil brightness. Int. J. Remote Sens. 11, 727–740. 10.1080/01431169008955053

[B23] Moreau L. Li Z. (1996). A new approach for remote sensing of canopy absorbed photosynthetically active radiation. *II.* Proportion of canopy absorption. Remote Sens. Environ. 55, 175–191. 10.1016/S0034-4257(95)00098-4

[B24] Muñoz J. D. Finley A. O. Gehl R. Kravchenko S. (2010). Nonlinear hierarchical models for predicting cover crop biomass using normalized difference vegetation index. Remote Sens. Environ. 114, 2833–2840. 10.1016/j.rse.2010.06.011

[B25] Musick H. B. Pelletier R. E. (1988). Response to soil moisture of spectral indexes derived from bidirectional reflectance in thematic mapper wavebands. Remote Sens. Environ. 25, 167–184. 10.1016/0034-4257(88)90099-5

[B26] Myneni R. B. Williams D. L. (1994). On the relationship between FAPAR and NDVI. Remote Sens. Environ. 49, 200–211. 10.1016/0034-4257(94)90016-7

[B27] Olofsson P. Laake P. E. V. Eklundh L. (2007). Estimation of absorbed PAR across scandinavia from satellite measurements: Part I: incident PAR. Remote Sens. Environ. 110, 252–261. 10.1016/j.rse.2007.02.021

[B28] Qi J. Chehbouni A. Huete A. R. Kerr Y. H. Sorooshian S. (1994). A modified soil adjusted vegetation index. Remote Sens. Environ. 48, 119–126. 10.1016/0034-4257(94)90134-1

[B29] Read J. J. Tarpley L. Mckinion J. M. Reddy K. R. (2002). Narrow-waveband reflectance ratios for remote estimation of nitrogen status in cotton. J. Environ. Qual. 31, 1442–1452. 10.2134/jeq2002.1442 12371160

[B30] Ridao E. Conde J. R. Nguez M. I. (1998). Estimating FAPAR from nine vegetation indices for irrigated and nonirrigated faba bean and semileafless pea canopies. Remote Sens. Environ. 66, 87–100. 10.1016/S0034-4257(98)00050-9

[B31] Rondeaux G. Steven M. Baret F. (1996). Optimization of soil-adjusted vegetation indices. Remote Sens. Environ. 55, 95–107. 10.1016/0034-4257(95)00186-7

[B32] Samanta A. Knyazikhin Y. Xu L. Dickinson R. E. Fu R. Costa M. H. (2012). Seasonal changes in leaf area of amazon forests from leaf flushing and abscission. J. Geophy. Res. 117, 1–13. 10.1029/2011JG001818

[B33] Skakun R. S. Wulder M. A. Franklin S. E. (2003). Sensitivity of the thematic mapper enhanced wetness difference index to detect mountain pine beetle red-attack damage. Remote Sens. Environ. 86, 433–443. 10.1016/S0034-4257(03)00112-3

[B34] Skianis G. A. Vaiopoulos D. Nikolakopoulos K. (2007). A probabilistic approach to the problem of assessing the efficiency of the transformed vegetation index. Remote Sens. Environ. 73, 461–480. 10.2495/SDP-V2-N4-461-480

[B35] Smith K. L. Steven M. D. Colls J. J. (2004). Use of hyperspectral derivative ratios in the red-edge region to identify plant stress responses to gas leaks. Remote Sens. Environ. 92, 207–217. 10.1016/j.rse.2004.06.002

[B36] Stenberg P. Rautiainen M. Manninen T. Voipio P. Smolander H. (2004). Reduced simple ratio better than NDVI for estimating LAI in finnish pine and spruce stands. Silva Fenn. 38, 3–14. 10.14214/sf.431

[B37] Steven M. D. (1998). The sensitivity of the OSAVI vegetation index to observational parameters. Remote Sens. Environ. 63, 49–60. 10.1016/S0034-4257(97)00114-4

[B38] Tan C. W. Samanta A. Jin X. L. Tong L. Ma C. Guo W. S. (2013). Using hyperspectral vegetation indices to estimate the fraction of photosynthetically active radiation absorbed by corn canopies. Int. J. Remote Sens. 34, 8789–8802. 10.1080/01431161.2013.853143

[B39] Turner D. P. Ritts W. D. Cohen W. B. Maiersperger T. K. Gower S. T. Kirschbaum A. (2005). Site-level evaluation of satellite-based global terrestrial gross primary production and net primary production monitoring. Remote Sens. Environ. 37, 27–49. 10.1111/j.1365-2486.2005.00936.x

[B40] Vepsäläinen M. Erkomaa K. Kukkonen S. Vestberg M. Wallenius K. Niemi R. M. (2004). The impact of crop plant cultivation and peat amendment on soil microbial activity and structure. Plant Soil. 264, 273–286. 10.1023/B:PLSO.0000047763.46795.cb

[B41] Wang B. L. Yang Y. Zheng S. H. Liu A. J. (2016). Study on estimation for FPAR of typical steppe based on the different vegetation index. Acta Agrestia Sinica. 24, 689–692.

[B42] Wang F. M. Huang J. F. Wang X. Z. (2009). Normalized difference ratio pigment index for estimating chlorophyll and cartenoid contents of in leaves of rice. Spectrosc. Spect. Anal. 29, 1064–1068. 10.3964/j.issn.1000-0593(2009)04-1064-05 19626904

[B43] Xiao Y. Zhao W. Zhou D. Gong H. (2014). Sensitivity analysis of vegetation reflectance to biochemical and biophysical variables at leaf, canopy, and regional scales. IEEE T. Geosci. Remote Sens. 52, 4014–4024. 10.1109/TGRS.2013.2278838

[B44] Yang F. Ren H. Li X. Hu M. Yang Y. (2014). Assessment of MODIS, MERIS, geov1 FPAR products over northern china with ground measured data and by analyzing residential effect in mixed pixel. Remote Sens. Basel. 6, 5428–5451. 10.3390/rs6065428

[B45] Zhang Q. Y. Middleton E. M. Margolis H. A. Drolet G. G. Barr A. A. Black T. A. (2009). Can a satellite-derived estimate of the fraction of PAR absorbed by chlorophyll (FAPAR chl) improve predictions of light-use efficiency and ecosystem photosynthesis for a boreal aspen forest? Remote Sens. Environ. 113, 880–888. 10.1016/j.rse.2009.01.002

[B46] Zhao P. J. Wang D. W. Huang C. Y. Ma Q. J. (2009). Estimation of cotton canopy fraction of photosynthetically active radiation (FPAR) and leaf area index (LAI) based on hyperspectral remote sensing data. Cotton Sci. 21, 388–393.

